# Ventilator-induced lung injury: from mechanisms to integrated clinical management

**DOI:** 10.3389/fmed.2026.1789457

**Published:** 2026-03-06

**Authors:** Liu-cun Li, Dan-hui Yang, Min Yang, Han Zhang, Lin Li, Xiang-yang Lu, Lin Wang, Hong Luo

**Affiliations:** 1 Department of Pulmonary and Critical Care Medicine, The Second Xiangya Hospital, Central South University, Changsha, Hunan, China; 2 Research Unit of Respiratory Disease, Central South University, Changsha, Hunan, China; 3 Clinical Medical Research Center for Pulmonary and Critical Care Medicine in Hunan Province, Changsha, Hunan, China; 4 Diagnosis and Treatment Center of Respiratory Disease in Hunan Province, Changsha, Hunan, China

**Keywords:** barotrauma, mechanical ventilation, ventilator, ventilator-induced lung injury, volutrauma

## Abstract

Currently, there are no definitive diagnostic criteria for Ventilator-induced lung injury (VILI), and the mechanisms underlying its development and progression remain incompletely understood. These mechanisms involve a complex interplay of factors, including barotrauma, volutrauma, atelectrauma, and biotrauma. Central to these issues are excessive lung tissue distension, the cyclic opening and closing of alveoli, and the activation and release of inflammatory mediators. Moreover, emerging concepts such as patient self-inflicted lung injury (P-SILI) and mechanical power have further expanded our understanding of VILI. These concepts underscore the critical roles of respiratory drive and mechanical energy transfer in the injury process. An in-depth analysis of the mechanisms underlying VILI suggests that its clinical prevention requires a dynamic and phase-specific strategy throughout the entire mechanical ventilation process. During the controlled ventilation phase, the primary focus should be on implementing a lung-protective ventilation strategy, which includes the use of low tidal volume and driving pressure, individualized positive end-expiratory pressure titration, prone positioning and extracorporeal life support to minimize pulmonary stress and strain. In the transition phase, attention should shift to modulating respiratory drive and ensuring optimal patient-ventilator synchrony to prevent P-SILI. Finally, during the weaning phase, emphasis should be placed on systematic assessment and spontaneous breathing trials to achieve safe liberation from mechanical ventilation. Here, we summarize the main mechanisms underlying VILI and outline prevention strategies to enhance understanding and management of this complication among clinical healthcare providers, ultimately to improve patient clinical outcomes.

## 1 Background

Ventilator-induced lung injury (VILI) refers to damage to lung tissue caused by mechanical ventilation, which arises from inappropriate ventilatory strategies or improper equipment utilization. Clinically, VILI may present with acute respiratory distress, persistent hypoxemia, and progressive deterioration in lung function. The occurrence of VILI poses substantial challenges in patient management and significantly worsens patient prognosis, making it a major clinical concern.

Currently, there are no definitive diagnostic criteria for VILI, as it does not arise from a single mechanism. Instead, different mechanisms may coexist, leading to diverse clinical presentations and imaging features. The mechanisms underlying the development and progression of VILI remain incompletely understood. These mechanisms involve a complex interplay of factors, including barotrauma, volutrauma, atelectrauma, and biotrauma. Central to these issues are excessive lung tissue distension, the cyclic opening and closing of alveoli, and the activation and release of inflammatory mediators. Moreover, emerging concepts such as patient self-inflicted lung injury (P-SILI) and mechanical power have further expanded our understanding of VILI. These concepts underscore the critical roles of respiratory drive and mechanical energy transfer in the injury process. Precise elucidation of VILI pathogenesis is fundamental to implementing stage-specific personalized prevention strategies throughout mechanical ventilation. This study aims to systematically summarize the main mechanisms of VILI and outline prevention strategies to enhance understanding and management of this complication among clinical healthcare providers, ultimately to improve patient clinical outcomes.

## 2 Main mechanisms of VILI

### 2.1 Ventilation at high lung volume/pressure

**Figure 1:**
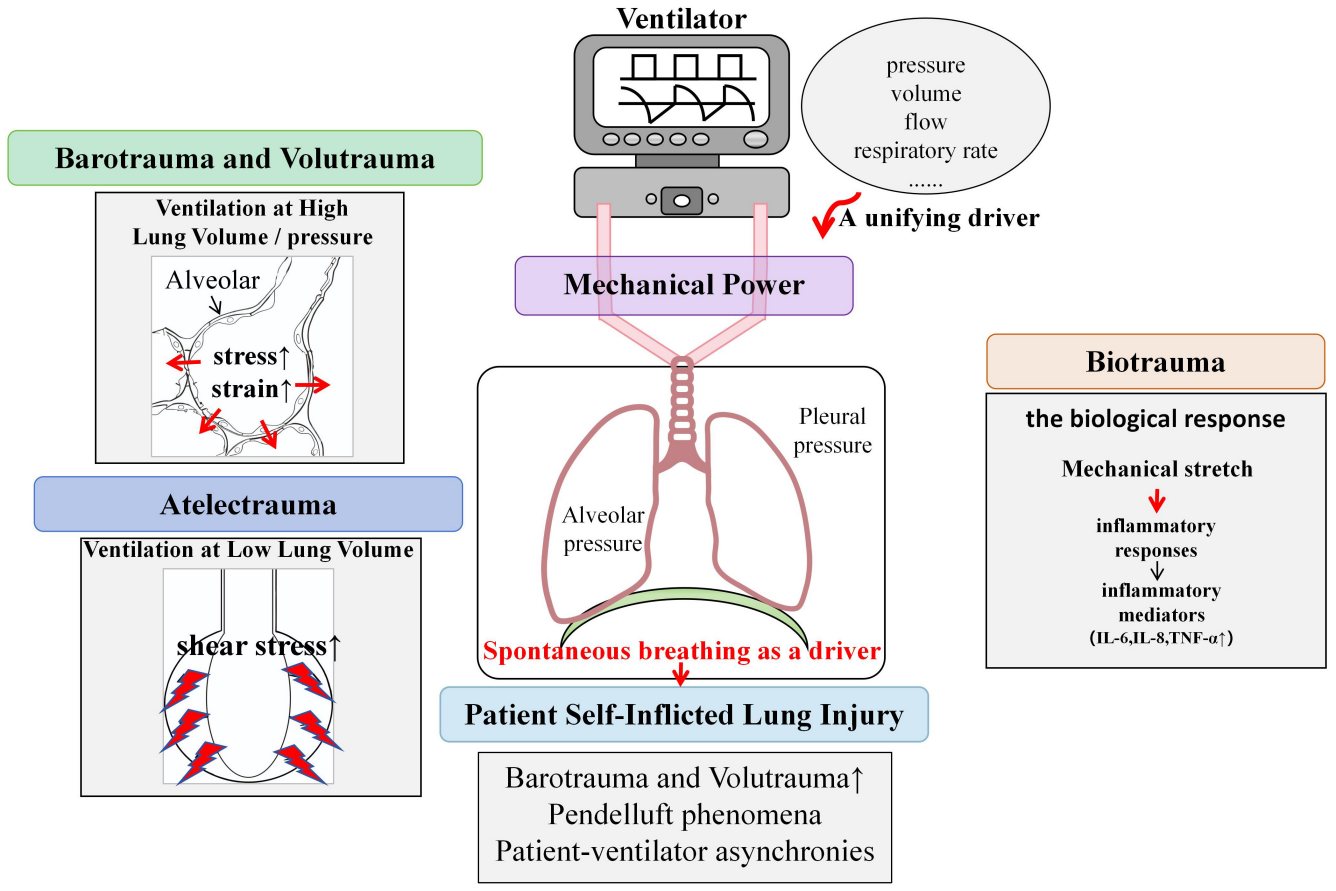
Main mechanisms of ventilator-induced lung injury.

Traditionally, two predominant theories have been proposed to explain the mechanisms of VILI: barotrauma and volutrauma. Some researchers suggested that high airway pressure is a key factor leading to lung injury, whereas others argued that high tidal volume may be a more important contributor to VILI (1) (Figure 1).

Why do these seemingly contradictory theories exist? The reason is that the pressure of barotrauma was previously defined as “airway pressure”, leading to the concept of barotrauma into question and subsequently prompting the proposal of volutrauma (1). However, when we redefine the “pressure” here, we find a close correlation between barotrauma and volutrauma. From a respiratory mechanics perspective, changes in pressure inevitably lead to corresponding changes in lung volume. Importantly, the pressure that determines changes in lung volume is not airway pressure, but rather transpulmonary pressure, which is the difference between alveolar pressure and pleural pressure (2). In a given patient, an increase in transpulmonary pressure may lead to an increase in tidal volume, resulting in excessive distension of alveoli and, in severe cases, alveolar rupture.

Let us revisit the classic research that introduced the concept of “volutrauma” and challenged the notion of “barotrauma.” In studies comparing two groups of animals ventilated at similar airway pressure, pulmonary edema was observed in the group with high tidal volume ventilation. In contrast, animals fitted with abdominal and thoracic binders-which mechanically restricted tidal volume-did not develop pulmonary edema (1). In the group of animals with low tidal volume, binding of the chest wall reduces its compliance, although the measured airway pressure is comparable, the transpulmonary pressure is significantly lower than that in the group with high tidal volume. Therefore, this finding does not negate the theory of “barotrauma”.

Consequently, it is now widely accepted that both barotrauma and volutrauma are closely related to excessive mechanical distension, which leads to excessive stress (i.e., transpulmonary pressure) on alveoli and excessive strain (strain = tidal volume/functional residual capacity) (3). On the basis of this understanding, lung-protective ventilation strategies should focus on limiting overall and local mechanical stress and strain by maintaining lower tidal volume and inspiratory transpulmonary pressure, thereby minimizing the risks of barotrauma and volutrauma.

### 2.2 Ventilation at low lung volumes

In addition to the mechanical distension discussed above, lung injury may also occur when ventilation is delivered at low (absolute) lung volumes, especially when there are marked heterogeneities in ventilation. This type of injury has been termed “atelectrauma.”

The mechanisms underlying atelectrauma mainly include: (1) the cyclic opening and closing of lung units generates significant increase of shear stress on terminal lung units, resulting in damaged alveolar epithelial cells; (2) lung collapse and the exudation of fluid within alveolar cavities, leading to decreased partial pressure of oxygen within alveoli; and (3) compression of alveolar cavities may inactivate pulmonary surfactant or displace it from the alveolar surface, thereby further exacerbating lung tissue damage.

Animal experiments conducted by Webb and Tiemey revealed that when high airway pressure is applied with zero end-expiratory pressure (PEEP), significant pulmonary edema occurs in the alveoli of rats. Conversely, when 10 cmH_2_O of PEEP is applied under the same airway pressure, no pulmonary edema is observed in the rats. These experimental results suggest that excessively low end-expiratory lung volume is also an important factor in VILI (4). Atelectrauma occurs when end-expiratory lung volume is critically low, causing repeated strain on the terminal airways, which readily triggers or amplifies VILI and complicates mechanical ventilation management. Mead et al. (5) reported that when a transpulmonary pressure of 30 cmH_2_O is applied to collapsed alveoli—restoring their lung volume to ten times of the pre-recruitment state, the surrounding normal alveoli are subjected to shear stress of 140 cmH_2_O.

Therefore, in clinical practice, it is essential to optimize mechanical ventilation strategies to avoid low end-expiratory lung volumes and to minimize the repeated opening and closing of terminal airways, thereby reducing the risk of atelectrauma.

### 2.3 Biological response: biotrauma

VILI can be caused by overdistension at high lung volume/pressure and collapse/reopening of airway units at low lung volumes. Mechanical stretch can induce the release of mediators associated with activation of the immune response, further adding to injury and potentially causing remote injury to other organs—this is termed “biotrauma” (6). Biotrauma is the biological response triggered by mechanical ventilation, whereby ventilatory forces activate pulmonary immune responses through various pathways, leading to inflammatory cell infiltration and inflammatory mediators release, causing direct or indirect lung tissue damage. Relevant studies have shown that increased levels of specific biological markers are closely associated with VILI, suggesting that biomarkers monitoring in clinical practice may aid in the early identification of VILI (7, 8).

It is currently understood that specific lung cells can detect the mechanical stimuli caused by excessive lung distension, and convert these stimuli into biochemical signals. These signals are transmitted into cells via specific signaling pathways, activating inflammatory cells within the lungs. This activation subsequently amplifies inflammatory responses, and drives the release of a large number of cytokines and inflammatory mediators that contribute to inflammatory injuries. Although various hypotheses have been proposed and extensively studied, the specific mechanisms underlying biotrauma remain unclear (9–12).

### 2.4 Spontaneous breathing as a driver: patient self-inflicted lung injury

For decades, research on VILI has focused on the direct pulmonary damage resulting from positive pressure ventilation. However, it is important to recognize that a substantial portion of mechanical ventilation is delivered in assisted modes during spontaneous breathing. In these settings, respiratory movement arises from the combined effects of positive pressure support and patient-driven inspiratory effort. Notably, the negative pleural pressure generated during spontaneous breathing can increase alveolar distention and regional lung stress, thereby contributing to lung injury. Under normal circumstances, pleural pressure and lung stress change uniformly during spontaneous breathing. However, in damaged lungs, spontaneous breathing can lead to more significant negative pleural cavity pressure in gravity-dependent areas. This understanding has led to a patient-related risk factor P-SILI, which refers to lung injury induced or exacerbated by the patient's respiratory efforts (13).

Currently, the main mechanisms of P-SILI include the following; (1) Excessive global lung stress: spontaneous breathing can markedly increase negative pleural cavity pressure, which directly increases transpulmonary pressure and subsequently tidal volume, resulting in barotrauma and volutrauma; (2) Excessive regional lung stress: in already injured lungs, excessive stress and strain are unevenly distributed during inflation. This can result in pendelluft phenomena, especially in patients with heterogeneous lung ventilation, thereby leading to lung injury (14); (3) Increased transvascular pressure: increased negative pleural cavity pressure enhances venous return and pulmonary blood flow perfusion, raising pulmonary vascular pressure, and potentially causing acute pulmonary edema and alveolar hemorrhage; and (4) Patient-ventilator asynchronies: particularly double triggering and reverse triggering can increase tidal volume and transpulmonary pressure, potentially resulting in pendelluft and further lung injury.

Introducing the concept of P-SILI into clinical practice provides healthcare professionals with a new perspective for understanding and preventing VILI. In recent years, a growing body of animal and clinical researches have supported the existence of the P-SILI mechanism and its potential harm. Many animal experiments have shown that even when tidal volume and plateau pressure are strictly controlled, intense spontaneous breathing can still cause lung injury through increased transpulmonary pressure, especially in patients with heterogeneous lung ventilation (15–17). Clinical studies have also indirectly supported the importance of P-SILI. Some studies have demonstrated that increases in respiratory drive and effort are closely related to the worsening of respiratory failure and increased need for intubation, even after adjustment for other variables (18). A retrospective study further revealed that increased spontaneous breathing drive is an important risk factor for progression of lung injury (19).

Therefore, protective ventilation strategies in clinical practice, such as low tidal volume, appropriate PEEP, and prone positioning, should be combined with carefully selected sedation and neuromuscular blockade to reduce P-SILI risk. Sedation and neuromuscular blockers can suppress excessive respiratory efforts to achieve protective ventilation. However, complete suppression of spontaneous breathing can cause respiratory muscles disuse atrophy and prolong the duration of mechanical ventilation, highlighting the need to optimize sedation strategies. Future research should focus on monitoring P-SILI and on developing more effective prevention strategies. Reliable bedside assessment of respiratory drive will provide important evidence for guiding clinical decisions and reducing P-SILI occurrence.

### 2.5 A unifying driver of VILI: mechanical power

In the clinical management of critically ill patients, especially those with ARDS, high mortality rates persist despite the use of lung-protective ventilation strategies that limit tidal volume and inspiratory pressure. This highlights the urgent need for new treatment strategies. Various factors of mechanical ventilation, such as pressure, volume, flow, and respiratory rate, may trigger VILI. However, the relative importance of each factor remains uncertain. In recent years, the concept of “mechanical power” (MP) has been proposed to explain how these variables contribute to the power delivered by the ventilator (20). MP represents the energy required by the ventilator to maintain ventilation and corresponds to the power delivered to the lungs. Lung injury caused by improper energy transfer during mechanical ventilation is termed energy injury. Unlike traditional individual parameters such as driving pressure (ΔP), MP integrates the contributions of pressure, volume, flow, and respiratory rate. It combines these into a single composite measure, thereby offering a more comprehensive assessment of the energy load imposed on the lung parenchyma (21). MP integrates traditional VILI assessment indicators and appears to be more effective in monitoring, assessing, and predicting VILI.

However, the standardization and clinical application of mechanical power still faces challenges. Gatinoni et al. (20) attempted to reformulate the respiratory equation into an energy equation, deriving a unified function that incorporates pressure, tidal volume, respiratory rate, inspiratory flow rate, lung compliance, and airway resistance. The resulting formula is as follows:

MP = {VT^2^ × (1/2 × Ers + RR × (1 + I/E)/(60 × I/E) × Rrs) + VT × PEEP} × RR × 0.098, where VT is the tidal volume, Ers is the elastance of the respiratory system, I/E is the inspiratory-to-expiratory time ratio, and Rrs is the resistance of the respiratory system.

However, this formula is relatively complex for clinical application. To improve its practicality, scholars have also attempted to simplify the formula to calculate the mechanical power using variables such as Ppeak, Vt, ΔP, and RR. The simplified MP formula shows good correlation and reliability with the results from the complex formula (22). For example, in volume control mode, the simplified formula is MP = 0.098 × RR × Vt × [Peak – $\frac{1}{2}$(Pplat – PEEP)], whereas in pressure control mode, it is MP = 0.098 × RR × Vt × (PEEP + ΔPinsp).

Although the effectiveness of MP has been confirmed, the specific MP threshold responsible for the development of VILI remains uncertain. Multiple animal and clinical studies suggests that high MP is closely related to the development of VILI. In some animal experiment models, lung injury worsens significantly when MP exceeds 12 J/min (23). Clinical studies have also revealed that when mechanical power exceeds 17 J/min, inpatient mortality increases significantly (24). Furthermore, higher MP continues to correlate with poorer ICU outcomes, even at low tidal volumes or driving pressures.

This leads to a critical question: which components of mechanical power are most related to the development of VILI (25). Some studies have included 4,549 ARDS patients to assess the impact of MP on mortality during mechanical ventilation and compared MP with other significant ventilation variables. The results found that driving pressure and respiratory rate are independent predictors of mortality. A simple model combining driving pressure and respiratory rate performed comparably to MP in predicting mortality, and offers greater practicality at the bedside (26). Consequently, a new combined parameter, “4DPRR” (i.e., 4 × ΔP + RR), has been proposed. However, some scholars believe that this model undermines the original physical value of the MP (27).

Ultimately, MP, reflecting the combined effects of multiple ventilator parameters such as pressure, volume, and respiratory rate, provides new ideas for VILI prevention. Although numerous preclinical and clinical studies have linked elevated MP to VILI, its translation to bedside practice remains constrained by the complexity of calculations, the absence of a standardized equation, and the lack of a well-defined optimal threshold. Furthermore, MP may vary substantially across different ventilation modes. For instance, Rietveld et al. demonstrated that in critically ill patients receiving controlled ventilation, volume-controlled ventilation (VCV) without an inspiratory pause resulted in the lowest MP, followed by pressure-controlled ventilation (PCV), whereas VCV with a 10% inspiratory pause was associated with the highest MP (28). Evidence from eight ARDSnet randomized controlled trials suggests that MP normalized to predicted body weight or respiratory system compliance offers better prognostic value than absolute MP, particularly in patients with moderate-to-severe ARDS (29). A recent study by Wu et al. further advances the field by proposing MP as a dynamic, physiology-based biomarker that may enhance the assessment of VILI risk and predict patients' responsiveness to interventions, such as prone positioning (30). Whether the MP can be used as a target for guiding mechanical ventilation implementation or as a warning tool still requires further validation. Additionally, the optimal MP threshold must consider various factors, such as age, sex, height, weight, degree of lung lesions, uniformity, FRC, compliance, and esophageal pressure. Developing standardized or dynamically adjusted MP may be a future research direction (31, 32).

## 3 VILI prevention strategies

**Figure 2:**
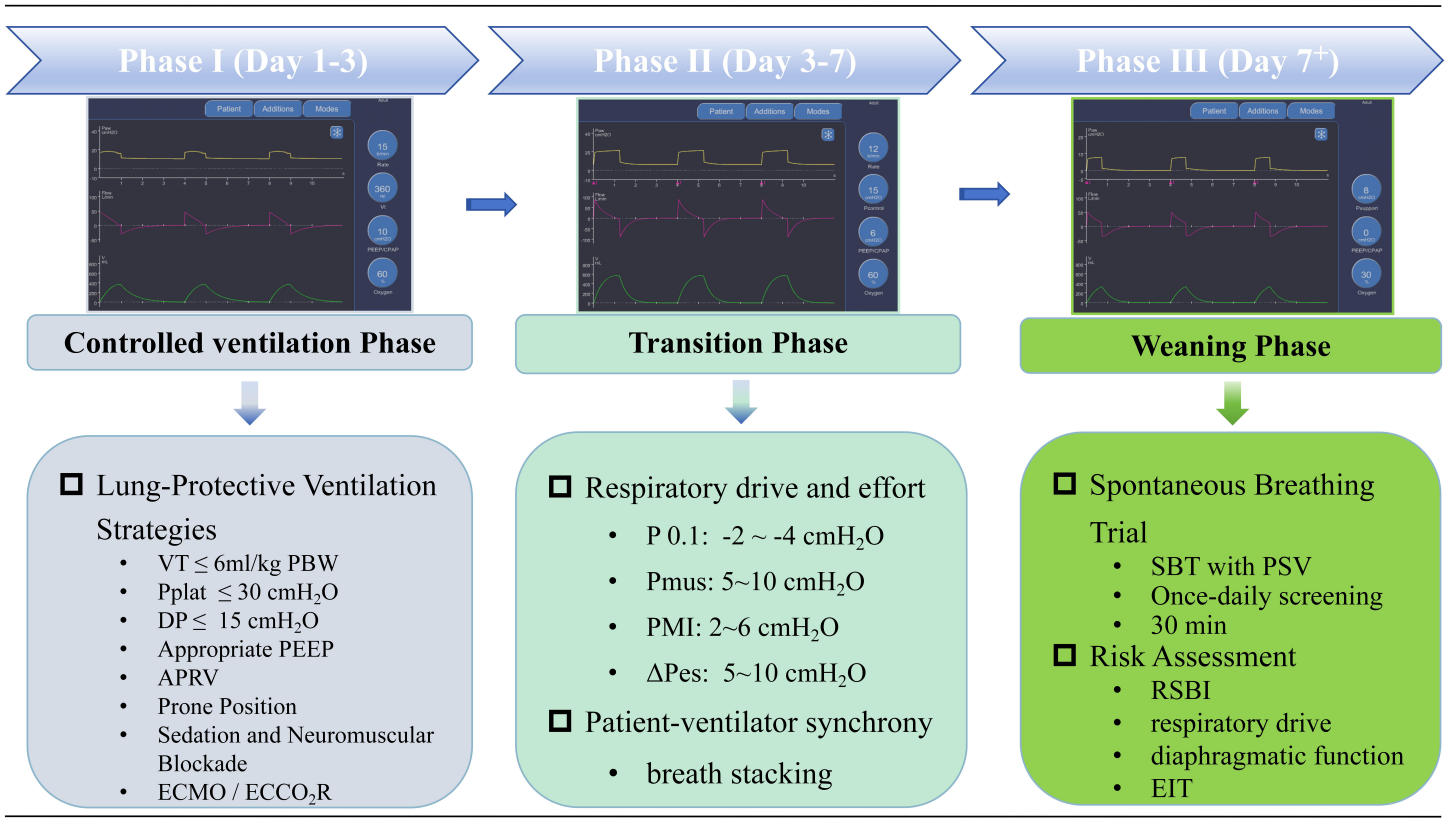
Ventilator-induced lung injury prevention strategies. VT, tidal volume; Pplat, plateau pressure; DP, driving pressure; PEEP, positive end-expiratory pressure; APRV, airway pressure release ventilation; ECMO, extracorporeal membrane oxygenation; ECCO_2_R, extracorporeal carbon dioxide removal; P0.1, airway occlusion pressure; Pmus, muscle pressure; PMI, pressure muscle index; ΔPes, esophageal pressure swing; SBT, spontaneous breathing trial; PSV, pressure support ventilation; RSBI, the rapid shallow breathing index; EIT, electrical impedance tomography.

Based on an in-depth analysis of the mechanisms underlying VILI, its clinical prevention requires a dynamic and phase-specific strategy that spans the entire process of mechanical ventilation. During the controlled ventilation phase, priority should be given to lung-protective ventilation strategy characterized by low tidal volume and optimized PEEP to minimize pulmonary stress and strain. In the transition phase, the focus shifts to modulating respiratory drive and ensuring patient-ventilator synchrony to avoid P-SILI (33, 34). Finally, during the weaning phase, emphasis should be placed on systematic assessment and spontaneous breathing trials to achieve safe liberation from mechanical ventilation (35) (Figure 2).

### 3.1 Controlled ventilation phase

During the initial phase of acute respiratory failure, when patients are deeply sedated and receiving passive ventilation, the primary objective is to prevent VILI through implementing Lung-Protective Ventilation Strategies. Current standards for Lung-Protective Ventilation Strategies include limiting tidal volume and airway pressure and using standard approaches to set PEEP, while evolving standards may focus on limiting driving pressure or mechanical power. Standard ventilatory adjuncts include prone positioning in moderate-severe ARDS and veno-venous extracorporeal life support in patients with severe hypoxemia or ventilatory difficulty.

#### 3.1.1 Low tidal volume ventilation and low driving pressure

The low-tidal-volume ventilation strategy (LTV) is a core component of lung-protective ventilation protocols. By restricting tidal volumes to 6 ml/kg predicted body weight (PBW) and maintaining plateau pressures (Pplat) below 30 cmH_2_O, this approach has demonstrated significant mortality reduction in major clinical trials (36, 37). However, although the 6 ml/kg PBW threshold provides a universally applicable safety baseline, it does not represent the optimal strategy for all patients (38).

How to determine whether the VT is appropriate? Setting VT on the basis of “milliliters per kilogram of PBW” to normalize VT to lung size might be misleading, as this approach overlooks the heterogeneity of ventilation in patients. It is more appropriate to adjust the VT according to the aerated lung region, which can be assessed by the compliance of the respiratory system (C_RS_) (39). Amato and colleagues posited that driving pressure (ΔP = VT/*C*_RS_) is the most precise “ventilator predictor” of outcomes in ARDS patients (40). Their findings suggested that maintaining ΔP below 15 cmH_2_O is associated with improved survival, whereas elevated ΔP—even with tidal volumes below 6 mL/kg PBW—correlates with poor clinical outcomes (41–43). This understanding underscores the necessity for dynamic tidal volume titration adjustment to minimize ΔP. Therefore, for patients who exhibit elevated ΔP despite LTV ventilation, further reduction of tidal volume to an ultra-low tidal volume strategy (4 ml/kg PBW) should be considered (44). This strategy aims to further reduce global lung stress and strain through enhanced ventilation restriction.

All ventilatory parameters are interdependent, and adjustments to one influence others. Therefore, when different VT targets are selected, their impacts on other ventilatory settings must be considered. The implementation of low-VT ventilation strategies inevitably introduces physiological challenges, particularly significant hypoventilation and severe hypoxemia. Although increasing RR and PEEP are common compensatory responses, these adjustments may paradoxically increase mechanical power and thereby increase the risk of VILI, potentially offsetting the benefits of reducing tidal volume (26). Therefore, mechanical ventilation management requires sophisticated, individualized titration of both tidal volume and respiratory frequency. Future research should prioritize identifying optimal ventilation patterns that simultaneously target ΔP and MP thresholds (40).

When conventional strategies fail to maintain safe levels of ΔP and MP, permissive hypercapnia (targeting pH > 7.25) may be considered. For refractory cases, extracorporeal life support including extracorporeal membrane oxygenation (ECMO) and extracorporeal CO_2_ removal (ECCO_2_R) can enable lung-protective ventilation while ensuring adequate gas exchange (45–47). The American Thoracic Society (ATS) updated guidelines conditionally recommend venovenous ECMO (VV-ECMO) for selected patients with severe ARDS, although based on low-certainty evidence (48). A meta-analysis including six studies indicated that in mechanically ventilated patients with severe ARDS, a driving pressure of 15 cmH_2_O may serve as an optimal threshold for initiating ECMO (49). This finding provides additional guidance for clinical decision-making regarding ECMO initiation. ECCO_2_R is an advanced life-support technique derived from ECMO technology. A meta-analysis of 14 studies involving 593 ARDS patients demonstrated that ECCO_2_R facilitates ultra-protective lung ventilation (50). However, its application carries risks of device-related complications, which necessitates careful patient selection. Currently, ECCO2R is considered to assist in implementing short-term lung-protective ventilation strategies in ARDS management, though it has not been shown to improve overall prognosis (51).

#### 3.1.2 Appropriate positive end-expiratory pressure settings

The primary role of PEEP is to keep alveoli open, improve compliance, and reduce atelectrauma caused by repetitive alveolar collapse and shearing. Insufficient PEEP can result in atelectrauma, whereas excessive PEEP may cause excessive alveolar distension (52). Therefore, appropriate PEEP settings are crucial for lung protection. Ideally, PEEP should be set as a “sweet spot” that maximizes alveolar recruitment while maintaining alveolar opening without inducing overdistention.

In patients with high recruitability, higher PEEP effectively recruits lung tissue and improves oxygenation. However, in those with low recruitability, the same PEEP level primarily acts on lung units that are already open, significantly increasing the risk of volutrauma (53). Static methods based on a single indicator (such as oxygenation) are inherently limited, as they cannot distinguish between “recruitable” and “overdistended” regions within an individual patient's lungs. Therefore, increasing PEEP based on predefined PEEP-FiO_2_ tables is suboptimal.

The optimal PEEP should be titrated based on a precise assessment of individual lung mechanics and recruitment potential. PEEP can be individualized by titrating to the point of best respiratory system compliance or by using advanced methods, including electrical impedance tomography (EIT) or esophageal pressure monitoring (54, 55). However, the application of these monitoring technologies in routine clinical practice faces several limitations. For instance, EIT equipment is expensive and provides limited image resolution (56). EIT also requires operator expertise for interpretation. Esophageal pressure monitoring involves the insertion of a specialized catheter, which may cause patient discomfort. Its measurements can be influenced by factors such as body position and pleural pressure variations, affecting stability and generalizability (57). Thus, future efforts should focus on simplifying procedures, reducing costs, and gathering additional evidence to support their routine use. Adjustments to mitigate high ΔP and MP, both possible drivers of VILI, may be further beneficial.

#### 3.1.3 Airway pressure release ventilation (APRV)

APRV delivers continuous positive airway pressure with brief, intermittent release phase, allowing partial lung volume release while permitting spontaneous breathing at higher pressure levels. In recent years, APRV has shown distinct advantages in managing ARDS and hypoxemia. A randomized controlled trial in COVID-19 ARDS patients found that APRV improved the PaO_2_/FiO_2_ ratio and static compliance compared to low tidal volume ventilation, though with a higher incidence of transient hypercapnia and no difference in ventilator-free days (58). Similarly, a multicenter propensity score-matched analysis found that intensive care unit survival, duration of mechanical ventilation, and complication rates were equivalent using APRV compared to conventional low tidal volume ventilation (59). Compared with conventional ventilation, APRV maintains safe and high airway pressure (Phigh) and for a prolonged time (Thigh), allowing for more complete recruitment of collapsed alveoli, thereby improving oxygenation (60). Additionally, the short release time (Tlow) of APRV ensures sufficient ventilation for CO_2_ clearance while limiting alveolar collapse, thereby preventing atelectasis and maintaining stable alveolar patency. This mechanism not only improves oxygenation but also reduces the risk of VILI (61). In particular, APRV offers protection against atelectrauma (62). A recent computational study using patient-derived digital twin models demonstrated that optimized APRV settings significantly reduced mean mechanical power and tidal alveolar recruitment/de-recruitment compared to pressure-controlled ventilation, providing novel mechanistic evidence for its lung-protective potential (63). Furthermore, electrical impedance tomography studies demonstrated that APRV optimizes the distribution of ventilation and perfusion, reduces lung heterogeneity, and potentially lower the risk of VILI (64). This is corroborated by a randomized controlled trial in patients with moderate to severe ARDS, which found that APRV, compared to low tidal volume ventilation, improved lung ventilation/perfusion matching and ventilation homogeneity (65). Therefore, APRV represents a promising protective ventilation strategy for mitigating VILI, but future prospective, large-scale studies are warranted for further validation (66).

#### 3.1.4 Prone position

Prone positioning, a strategy that has been used for many years, is now recommended for patients with moderate-to-severe ARDS (67). It is easy to perform, does not requires expensive equipment or medications, adds no additional medical costs, and carries low risk of fatal complications. Prone positioning promotes the recruitment of collapsed alveoli in dependent lung regions, improves ventilation-perfusion ratios, and enhances respiratory system compliance (68).

Prone positioning ventilation improves pulmonary homogeneity, reduces the risk of alveolar overdistension and collapse, thereby mitigating barotrauma and atelectrauma. Concurrently, it enhances respiratory system compliance, which contributes to lung protection by potentially reducing driving pressure and mechanical power (69).

Studies have confirmed that prone positioning can significantly reduce mortality in moderate to severe ARDS patients, with better effects when it is applied early ( ≤ 7 days) (70, 71). However, its complications, such as pressure injuries, must be carefully considered (72). Additionally, the alveolar recruitment achieved with prone positioning is time-dependent, and each prone positioning ventilation should last at least 12–16 h. Some scholars have proposed extended prone positioning (EPP) in recent years, which is typically defined either as avoiding unnecessary turning or as maintaining the position for more than 24 h. In comparison with traditional prone positioning (lasting 16 h), EPP may improve oxygenation and reduce in-hospital mortality (73). However, further research is still required to confirm the optimal duration for implementing EPP and its underlying mechanisms.

#### 3.1.5 Sedation and neuromuscular blockade

For critically ill patients in the early phase of intubation (within 1–3 days), sedation and neuromuscular blocking agents (NMBAs) play a vital role in facilitating lung-protective ventilation strategies, enhancing patient-ventilator synchrony, and improving ventilation and oxygenation. In patients with ARDS receiving mechanical ventilation, NMBA administration reduces the work of breathing and patient-ventilator asynchrony, which may positively influence clinical outcomes (74, 75). However, prolonged use of NMBAs is associated with risks such as neuromuscular weakness and often necessitates deep sedation, which may itself lead to adverse effects (76).

In the recent ROSE trial, patients with moderate-to-severe ARDS were randomized to a 48-h continuous NMBA infusion with deep sedation (intervention group) or to a usual-care approach. The usual-care approach omitted routine neuromuscular blockade and used lighter sedation targets (control group). The trial found no significant difference in mortality at 90 days (77). In contrast, the ACURASYS trial reported a protective effect on 90-day outcomes (78). Several notable differences exist between the two large studies (77, 78). The ROSE trial had a lower rate of prone positioning ventilation (15.8% vs. 44.8%), higher PEEP, and lighter sedation in the control group than the ACURASYS trial. A 2025 secondary analysis of the ROSE trial assessed whether early NMBA use affected mortality based on baseline physiology and lung injury biomarkers, including respiratory system elastance (Ers). The findings suggest high Ers may define a patient phenotype that benefits from early NMBA use (79). However, its stability and suitable patient populations are not fully established and require further investigation.

For early severe ARDS (PaO_2_/FiO_2_ < 100 mmHg with symptom onset < 48 h), the 2024 updated guidelines from the ATS recommend short-term use of NMBAs to improve patient-ventilator synchrony and oxygenation (conditional recommendation, low certainty of evidence) (48). Guidelines from the European Society of Intensive Care Medicine (ESICM) state that routine prolonged NMBA infusion should not be used (moderate level of evidence) (80). Caution is advised due to risks such as myopathy. Therefore, take care when using neuromuscular blockers to avoid long-term effects and complications (81).

### 3.2 Transition phase

As soon as the patient's condition improves, every mechanically ventilated patient must transition from passive, controlled ventilation to assisted ventilation and, ultimately, to weaning. During this phase, the major risk for lung injury arises from excessive global and regional pulmonary stress and from patient-ventilator asynchrony associated with spontaneous breathing (34). A successful transition can restore spontaneous breathing, mitigate VILI, and improve clinical outcomes. In clinical practice, the transition from controlled to assisted ventilation requires clinicians to carefully balance the physiological benefits of spontaneous breathing with the risk of excessive inspiratory efforts and increased lung stress and strain.

#### 3.2.1 Assessment and control of respiratory drive and effort

The consequences of high respiratory drive and effort are considerable (82). In patients on mechanical ventilation, insufficient or excessive respiratory drive and effort were associated with higher ICU mortality and lower rate of ICU discharge, particularly in those with more severe oxygenation impairment (83, 84).

Monitoring and regulating respiratory drive and effort at the bedside are fundamental steps in preventing VILI during the transition phase. Methods for assessing respiratory drive/effort mainly include the following (Table 1):

1. Clinical signs such as accessory inspiratory muscle use, expiratory muscle contraction, diaphoresis, tachycardia, nose flaring, intercostal retraction and dyspnoea indicate excessive respiratory drive. However, these indicators are not quantifiable.
2. Indicators related to respiratory drive and effort include airway occlusion pressure (P0.1) (85), airway occlusion pressure (ΔPocc) (86), esophageal pressure (Pes) (87, 88), and diaphragm ultrasound (89).
3. Diaphragm electrical activity offers a quantitative assessment of central drive to the diaphragm and provides a more intuitive basis for evaluating respiratory drive (90).

**Table 1:** Bedside monitoring indicators for the respiratory drive and drive.

**Indices**	**Clinical significance and recommendation**	**Level of evidence^c^**
Clinical symptoms	Respiratory distress^**a**^	C
P0.1	Normal values are between −2 and −4 cmH_2_O	A
ΔPocc^b^	Predicted Pmus = −3/4 × Pocc Predicted ΔPL, dyn = (Ppeak − PEEP) – 2/3 × Pocc	B
ΔPes	Normal values are between 5 and 10 cmH_2_O	A
ΔPL	Possibly keep below 10–12 cmH_2_O	A
End inspiratory PL	Possibly keep below 20–25 cmH_2_O	B
Pmus	Normal values are between 5 and 10 cmH_2_O	B
PMI	Normal values are between 2 and 6 cmH_2_O	C
PTPes	Normal values are around 100 cmH_2_O s min^−1^	B
Work of breathing	Normal values are around 0.35 or 2.4 J min^−1^	B

P0.1, airway occlusion pressure; Pes, esophageal pressure; ΔPes, esophageal pressure swing; PL, transpulmonary pressure; ΔPL, transpulmonary driving pressure; Pmus, Muscle pressure; PMI, Pressure Muscle Index; PTPes, esophageal pressure (Pes)-time product.

^a^Clinical signs and symptoms indicating respiratory distress are considerable, including accessory inspiratory muscle use, expiratory muscle contraction, diaphoresis, tachycardia, nose flaring, intercostal retraction and dyspnea.

^b^Clinicians can use Pocc to estimate respiratory muscle effort (Pmus) and ΔPL, dyn. An estimated Pmus > 15 cmH_2_O and an estimated ΔPL, dyn > 20 cmH_2_O indicate that respiratory effort and dynamic lung stress, respectively, are likely excessive.

^c^Level A strong evidence from multiple high-quality randomized controlled trials or meta-analyses. Level B: moderate evidence from single randomized trials, observational studies, or high-quality retrospective analyses. Level C: limited evidence based on expert consensus, case reports, or pathophysiological rationale.

Factors that increase respiratory drive include hypercapnia, hypoxemia, metabolic acidosis, inflammatory responses, lung atelectasis, and patient-ventilator asynchrony (91). Different causes require different management strategies, including sedation, analgesia, neuromuscular blockers, respiratory support, prone positioning, ECCO_2_R, and ECMO.

#### 3.2.2 Ensuring patient-ventilator synchrony

Patient-ventilator synchrony is critical during mechanical ventilation, as asynchrony may worsen lung injury through multiple mechanisms (92). As spontaneous breathing resumes, patient-ventilator asynchrony is frequently observed. Breath stacking (e.g. double triggering) is particularly common under in the presence of high inspiratory effort (93). This asynchrony can lead to excessive tidal volumes and elevated transpulmonary pressures, significantly increasing the risk of VILI.

The mechanisms of injury differ by ventilation mode: in VCV, regional overdistention may occur as a result of gas trapping, whereas in PCV, vigorous inspiratory efforts can resulting in stress concentration and atelectrauma (94). When asynchrony such as double triggering is detected, it should be considered as a clinical warning sign, prompting immediate evaluation of respiratory drive, esophageal pressure dynamics, and respiratory mechanics. Ventilator modes and support levels should be promptly adjusted to restore synchrony and minimize the risk of VILI.

### 3.3 Weaning phase

Early liberation from mechanical ventilation represents the most direct and fundamental strategy to reduce the incidence of VILI (95). Successful liberation from mechanical ventilation depends on the accurate assessment of patient readiness, adherence to an evidence-based weaning protocol, and a multidimensional evaluation of the risk for weaning failure. Systematic daily assessment of a patient's readiness for liberation from mechanical ventilation is crucial (96). This evaluation typically involves screening for improvement in the underlying disease, respiratory mechanics and oxygenation, and other clinical indicators. The spontaneous breathing trial (SBT) is the gold standard for assessing weaning readiness (97).

#### 3.3.1 Which technique?

SBTs are most commonly performed using low levels of pressure-support ventilation (PSV), or with a *T*-piece, in which the patient is disconnected from the ventilator and receives additional oxygen (98). The official American College of Chest Physicians/American Thoracic Society Clinical Practice Guideline conditionally recommends that for acutely hospitalized adults ventilated for more than 24 h, the initial SBT should be conducted with inspiratory pressure augmentation (5–8 cm H_2_O) rather than without it (i.e., with a *T*-piece or CPAP) (99). This recommendation is supported by evidence demonstrating that an SBT conducted with pressure support is comparably effective to a *T*-piece trial in predicting successful extubation and may facilitate the liberation process without increasing reintubation risk (100, 101). Furthermore, SBT using PSV offer practical advantages in the ICU setting, as they do not require disconnecting the patient from the ventilator circuitry. Another technical consideration is whether PEEP should be applied during a PSV-based SBT. Current evidence suggests that a trial using PSV without PEEP is easier for patients to pass than a T-piece trial and can safely facilitate extubation. Therefore, PSV without PEEP should be considered as the first option for assessing extubation readiness in ICUs (35). Whether an even less demanding SBT with PSV and PEEP is safe requires further research.

#### 3.3.2 How often?

Recent evidence indicates that among critically ill adults receiving invasive mechanical ventilation for over 24 h, neither the screening frequency (once-daily vs. more frequent) nor the SBT technique (pressure-supported vs. *T*-piece) had significant effect on the time to successful extubation. An unexpected interaction between screening frequency and SBT technique required pairwise contrasts, which revealed that more frequent screening (vs. once-daily screening) and pressure-supported SBT increased the time to successful extubation [HR, 0.70 (95% CI, 0.50–0.96); *P* = 0.02] (102). These results suggest that once-daily screening may be more appropriate.

#### 3.3.3 How long?

Extubation success and other clinically important outcomes were comparable between short (30 min) and long (120 min) SBT. A 30-min SBT may therefore be acceptable in this context. A shorter SBT duration can also expedites the extubation process (103).

#### 3.3.4 Assessment of weaning failure risk

Although early liberation from mechanical ventilation is of paramount clinical significance, it requires careful risk-benefit balancing to avoid premature or rushed extubation that may lead to reintubation. Reintubation was associated with a significant higher in-hospital and ICU mortality (104). Elevated reintubation rates, frequently associated with post-extubation respiratory failure, may consequently increase the risk of VILI. Therefore, extubation decisions should be based on comprehensive, multidimensional assessment rather than on procedural speed alone (105). Prudent evaluation of weaning timing is essential to prevent increasing reintubation risks through premature extubation.

Early identification of high-risk populations—utilizing predictors including the rapid shallow breathing index (RSBI), respiratory drive, diaphragmatic function, and EIT—derived parameters—is recommended to reduce the incidence of reintubation (106, 107). The RSBI, calculated as the ratio of respiratory rate to tidal volume, is a commonly used predictor. Evidence indicates that an RSBI > 105 breaths/L/min is associated with weaning failure, while a value below 105 breaths/L/min indicates a higher likelihood of successful weaning (108). Failure during a SBT often stems from an imbalance between respiratory muscle capacity and load. In high-risk patients, an SBT-induced excessive increase in inspiratory effort is a key mechanism linked to extubation failure (109). To assess the primary respiratory muscle, diaphragm ultrasound has emerged as a practical bedside tool to evaluate function and exclude significant dysfunction as a contributor to weaning failure. A diaphragm thickening fraction (TFdi) < 30%−35% and diaphragm excursion (DE) < 10–15 mm have been shown to be predictive of weaning failure (110). Beyond global indices and muscle-specific assessment, research into novel monitoring aims to provide deeper physiological insight. EIT is a non-invasive imaging modality and a promising tool to gain physiological insight into the weaning process (111). EIT has the potential to assess changes in ventilation distribution and quantify the inhomogeneity of the lungs during the SBT (107). High lung inhomogeneity was found during SBT failure. Integrating these multidimensional assessments is crucial for a personalized approach to liberating patients from mechanical ventilation.

In summary, VILI is a major challenge in mechanical ventilation treatment, and its complex mechanisms and diverse clinical manifestations continue to draw researchers' attention. From the traditional concepts of barotrauma, volutrauma, atelectrauma, and biotrauma to the newly proposed concepts of P-SILI and mechanical power, in-depth researches into these mechanisms have greatly deepened our understanding of VILI. Evidence indicates that lung injury is not attributable to a single factor but rather results from the interaction of multiple factors. These findings provide a foundation for developing more comprehensive prevention and treatment strategies in clinical practice.

The prevention of VILI necessitates an integrated, dynamic strategy tailored to the patient's evolving physiological state throughout the course of mechanical ventilation. During controlled ventilation, a foundational lung-protective approach—emphasizing low tidal volume, limiting driving pressure, individualized PEEP titration, and prone positioning—effectively reduces global stress and strain. As patients transition to spontaneous breathing, close monitoring and appropriate modulation of respiratory drive, combined with optimization of patient-ventilator synchrony, are critical to prevent patient self-inflicted lung injury. In the liberation phase, structured weaning protocols incorporating spontaneous breathing trials and multidimensional risk assessment are critical to ensuring safe extubation. Across all phases, the central principle is personalized ventilation management guided by advanced monitoring and physiological targets to mitigate VILI risk while promoting successful weaning. Future efforts should focus on developing real-time monitoring technologies and precision-based protocols to dynamically assess patients' lung stress and energy-related injury risk. Recent studies have identified EIT as a potential monitoring tool for VILI (112). As a bedside, non-invasive, and continuous imaging modality, EIT can assess regional ventilation distribution and ventilation/perfusion (V/Q) matching, thereby enabling precise evaluation of lung heterogeneity and furnishing critical information for clinical monitoring and prevention of VILI (113). In retrospective analyses of patients with ARDS, the degree of *V*/*Q* mismatch quantified by EIT correlates with disease severity and therapeutic response (114). Prospective research by Spinelli et al. further supports these findings, demonstrating significant associations between *V*/*Q* mismatch, physiological markers of lung injury, and established ARDS biomarkers (115). Collectively, these observations highlight the potential of EIT as a valuable modality for monitoring VILI in mechanically ventilated patients.

Future research should focus on several key directions: first, further elucidating the mechanisms of VILI and developing more precise clinical assessment tools; second, advancing individualized ventilation strategies tailored to different patient populations; and finally, enhancing the application of advanced monitoring technologies, such as EIT and Ptp monitoring, in the prevention of VILI.

In conclusion, both the investigation and prevention of VILI continue to advance. Through ongoing mechanistic studies and refinement of clinical strategies, we can strengthen the prevention and control of VILI, improve patient outcomes, and advance critical care practice toward more precise and individualized approaches. This represents not only an improvement in current mechanical ventilation practice but also a meaningful shift in the overall therapeutic approach to critically ill patients.
